# Supplementary material for: The interactome of intact mitochondria by cross-linking mass spectrometry provides evidence for coexisting respiratory supercomplexes
Source: Mol Cell Proteomics. 2017 Dec 8;17(2):216–32. doi: 10.1074/mcp.RA117.000470 (PMC5795388; doi:10.1074/mcp.RA117.000470)
Supplement: Supplemental Data [file supp_RA117.000470_133922_0_supp_23978_fzffwf.zip › spectra_annotation/mito_DR_spectra_annotation/243-2-6-1-2-1.pdf]

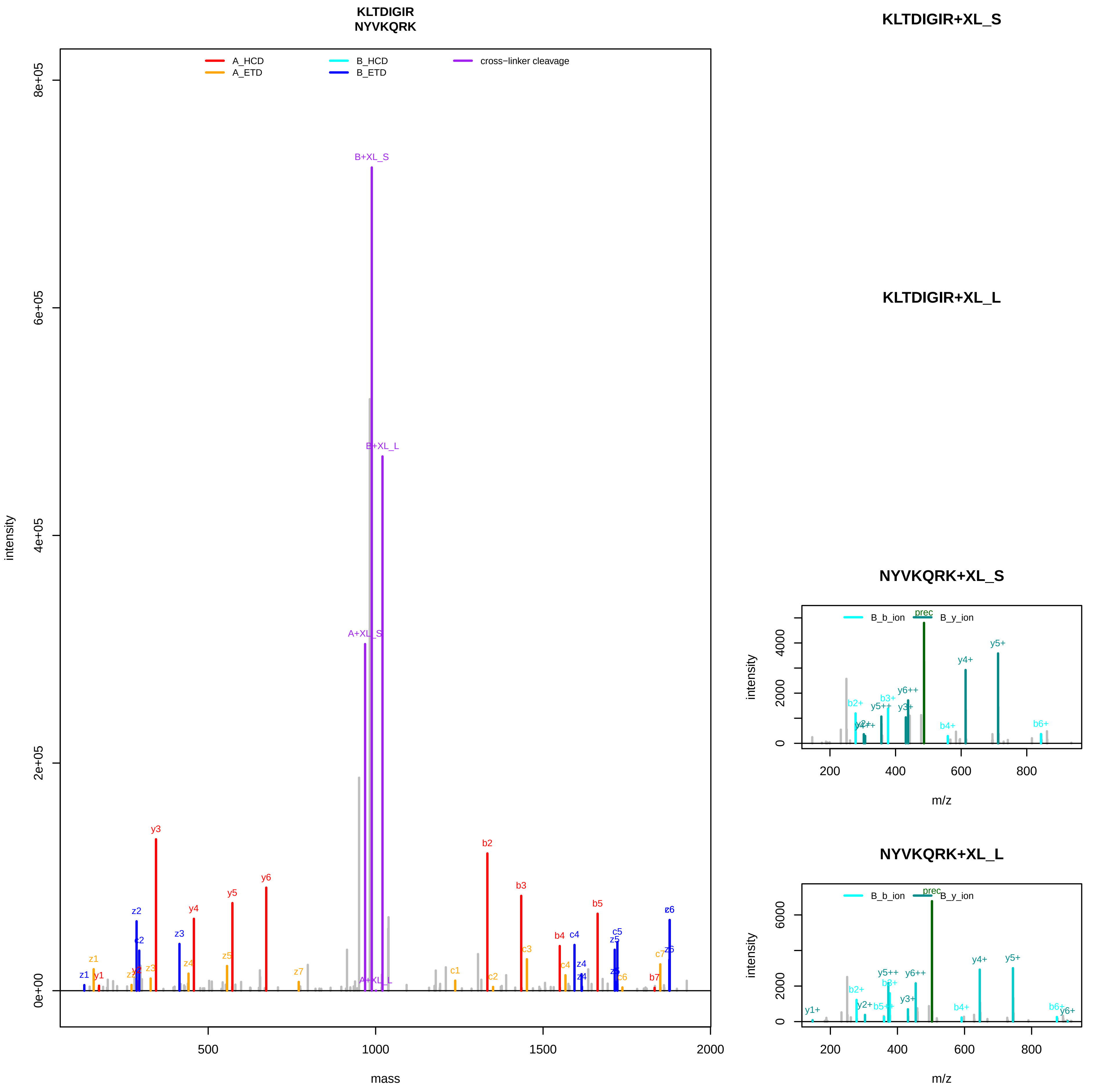
KLTDIGIR
NYVKQRK
A_HCD
A_ETD
B_HCD
B_ETD
cross−linker cleavage
intensity
mass
B+XL_S
B+XL_L
A+XL_S
A+XL_L
y3
y4
y5
y6
b2
b3
b4
b5
b7
z1
z2
z3
z4
z5
z6
z7
c1
c2
c3
c4
c5
c6
c7
KLTDIGIR+XL_S
KLTDIGIR+XL_L
NYVKQRK+XL_S
NYVKQRK+XL_L
B_b_ion
B_y_ion
prec
m/z
y1+
y2+
y3+
y4+
y5+
y5++
y6++
y6+
b2+
b3+
b4+
b5++
b6+
